# Resected lymph nodes and survival of patients with esophageal squamous cell carcinoma: an observational study

**DOI:** 10.1097/JS9.0000000000000436

**Published:** 2023-05-24

**Authors:** Kexun Li, Xuefeng Leng, Wenwu He, Kunyi Du, Changding Li, Kun Liu, Chenghao Wang, Guangyuan Liu, Zhiyu Li, Longlin Jiang, KunZhi Li, Qiang Zhou, Jialong Li, Xi Luo, Xin Nie, Simiao Lu, Haojun Li, Qiang Fang, Wenguang Xiao, Yongtao Han, Lin Peng

**Affiliations:** Division of Thoracic Surgery, Sichuan Cancer Research Center for Cancer, Sichuan Cancer Hospital and Institute, Sichuan Cancer Center, Afliated Cancer Hospital of University of Electronic Science and Technology of China, Chengdu, Sichuan, People's Republic of China

**Keywords:** esophageal squamous cell carcinoma, esophagectomy, lymphadenectomy, positive lymph nodes

## Abstract

The incidence and mortality of esophageal cancer are high. Therefore, the authors aimed to investigate how the number of dissected lymph nodes (LNs) during esophagectomy for esophageal squamous cell carcinoma impacts overall survival (OS), particularly that of patients with positive LNs.

**Materials and methods:**

Data from 2010 to 2017 were obtained from the Sichuan Cancer Hospital and Institute Esophageal Cancer Case Management Database. Participants were divided into two groups: patients with negative lymph nodes (N0) and patients with positive lymph nodes (N+). The median number of resected LNs during surgery was 24; therefore, patients with 15–23 and those with 24 or more resected LNs were assigned to subgroups A and B, respectively.

**Results:**

After a median follow-up of 60.33 months, 1624 patients who underwent esophagectomy were evaluated; 60.53 and 39.47% had a pathological diagnosis of N+ or N0, respectively. The median OS was 33.9 months for the N+ group; however, the N0 group did not achieve the median OS. The mean OS was 84.9 months. In the N+ group, the median OS times of subgroups A and B were 31.2 and 37.1 months, respectively. The OS rates at 1, 3, and 5 years were 82, 43, and 34%, respectively, for subgroup A of the N+ group; they were 86, 51, and 38%, respectively, for subgroup B of the N+ group. Subgroups A and B of the N0 group exhibited no statistically significant differences.

**Conclusion:**

Increasing the number of LNs harvested during surgery to 24 or more could improve the OS of patients with positive LNs but not that of patients with negative LNs.

## Graphical abstract

**Figure:**
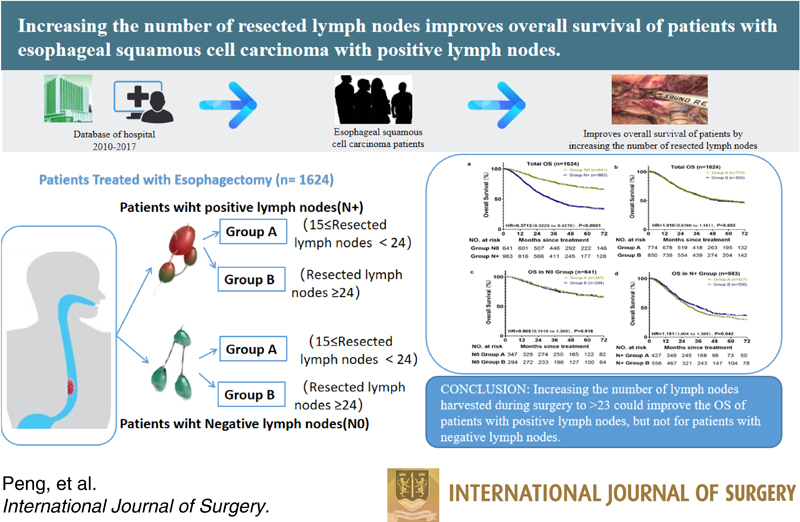

## Introduction

Highlights

- Esophageal squamous cell carcinoma is the main occurring subtype in China.
- Overall survival is associated with an increased number of resected lymph nodes.
- An esophagectomy was mainly performed through a right transthoracic procedure.
- The pathological N stage is an independent prognostic factor for overall survival.

According to the Systematic Analysis for the Global Burden of Disease Study, the numbers of new cases and deaths of esophageal cancer in 2019 were 535 000 and 498 000, respectively, and its incidence and mortality were ranked high among the 29 tumor categories investigated^1^. Esophageal cancer is the fourth primary cause of cancer-related death and has the sixth highest incidence in China^2^. Esophageal squamous cell carcinoma (ESCC) is the main subtype in China, accounting for ~90% of cases^3^. Radical resection of esophageal cancer is recommended for all resectable cases. Surgery with radiotherapy and chemotherapy comprise the cornerstone treatment for localized ESCC^4^.

In recent years, the CROSS and CheckMate-577 trials have enabled remarkable achievements. The CROSS trial indicated that the median disease-free survival of patients undergoing surgery alone was 11.6 months; however, that of patients with squamous cell carcinoma treated with neoadjuvant chemoradiotherapy was 74.7 months. CheckMate-577 revealed that the median disease-free survival of patients treated with neoadjuvant chemoradiotherapy was 11 months for the squamous cell carcinoma subgroup; however, it was 22.4 months for the immunomaintenance therapy subgroup^5,6^. After the randomized, controlled CROSS trial, the CROSS study group launched the TIGER study, which demonstrated that the establishment of an optimal surgical strategy for esophageal cancer patients has been increasingly emphasized by Western surgeons^7,8^. Several studies have confirmed that lymph node (LN) metastasis (LNM) is an important factor impacting overall survival (OS) associated with esophageal cancer^9–11^. In recent years, Chinese researchers have conducted studies of LN dissection; however, the sample sizes were small^12–14^.

In this study, we evaluated the impact of the number of resected LNs (RLNs) on the OS of patients with thoracic ESCC (TESCC) who underwent esophagectomy. In addition, we determined the effects of different numbers of RLN segments during surgery on the OS of patients with positive LNs (N+) and those with negative LNs (N0).

## Materials and methods

The data were obtained from the database at our institution. We performed a retrospective analysis of patients with esophageal cancer from January 2010 to December 2017. The study was approved by the Ethics Committee for Medical Research and New Medical Technology of our hospital. The research has been reported in line with the Strengthening the Reporting of Cohort Studies in Surgery (STROCSS) guidelines^15^, Supplemental Digital Content 1, http://links.lww.com/JS9/A571.

The retrieved data included demographic and pathological information, such as sex, age, T stage, N stage, tumor–node–metastasis (TNM) stage, tumor location (upper, middle, or lower thoracic), tumor grade, nerve invasion, central LNM, and radical resection. Esophagectomy was mainly performed using a right transthoracic procedure with two-field or three-field LN dissection; the surgical approaches were dependent on the patient’s characteristics and the surgeon’s discretion. The disease stage was classified according to the American Joint Committee on Cancer eighth edition of the TNM system. Patients were followed up once every 3 months for the first 2 years; thereafter, they were followed up once every 6 months for 3 to 5 years. A total of 2957 patients with TESCC treated with esophagectomy were identified from January 2010 to December 2017. There were three inclusion criteria: esophagectomy was performed; the tumor was located in the thoracic esophagus; and the pathology results confirmed squamous cell carcinoma. The exclusion criteria were as follows: less than 15 RLNs were resected during surgery; other malignant tumors were present; pathological T stage (Tis/T1a/T4/M1); preoperative treatment was performed; and required data were missing. The OS was calculated from the month and year of surgery to death or the last follow-up evaluation in March 2021. For survival outcomes, we used the median as far as possible; when the medians were not achieved, means were used.

Patients were divided into two groups. Patients with N0 according to the pathological results were assigned to the N0 group. Patients whose pathological results showed N+ were assigned to the N+ group. Because the median number of RLNs during surgery was 24, patients with 15–23 RLNs were assigned to subgroup A, and those with 24 or more RLNs were assigned to subgroup B. The TNM stages were used to compare clinical outcomes and survival data.

## Theory/calculation

### Statistical analysis

Class variables are expressed as percentages. We calculated the results using *χ* ^2^ or Fisher precision tests. Independent OS-related risk factors were identified by single-variable and multivariable logistic regression analyses; hazard ratios and 95% CIs were calculated. The impact of all baseline covariates on the results was assessed using the Cox proportional disaster hazards regression model. The observation system was evaluated using Kaplan–Meier curves, and the results of the logarithmic grade tests were compared to describe the median at specific time points as the 95% CI. *P*<0.05 was considered statistically significant. All analyses were performed using SPSS version 23.0 (Chicago).

## Results

### Patient characteristics

**Figure 1:**
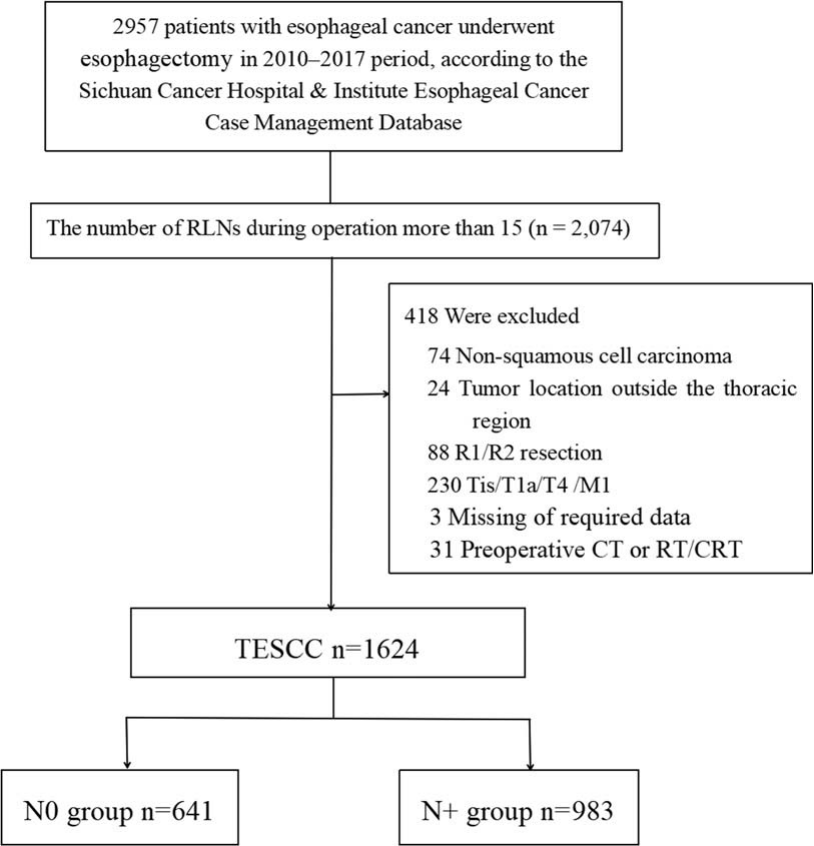
CONSORT diagram showing the patient selection process. TESCC, thoracic esophageal squamous cell carcinoma; N0, patients with negative lymph nodes according to the pathological results; N+, patients with positive lymph nodes according to the pathological results.

From January 2010 to December 2017, the data of 1624 patients were retrieved and retrospectively analyzed; of these, 641 (39.5%) and 983 (60.5%) had pathological N0 and N+, respectively. Males comprised 83.0% (1348/1,624), and females comprised 17.0% (276/1624) of the patients. More than half of them (60.5%; 983/1,624) exceeded pathological stage III. There were 1473 patients who underwent two-field LN dissection, 88 patients who underwent three-field LN dissection, and 63 patients who underwent two-field LN dissection combined with unilateral neck dissection. Among these patient groups, the median numbers of dissected LNs were 23, 39, and 36, respectively. A total of 3683 (8.6%) supraclavicular LNs were dissected. Moreover, 259 of 3683 (7.0%) LNs were positive. There were 23 608 (55.3%) mediastinal (thoracic) LNs, and 2030 of 23 608 (8.5%) LNs were positive. There were 15 397 (36.1%) abdominal LNs. Moreover, 1343 of 15 379 (8.7%) LNs were positive. During this study, 641 participants comprised the N0 group, and 983 comprised the N+ group (Fig. 1). The clinicopathological and pathological characteristics of the N0 and N+ groups are presented in Table 1. Subgroup B of the N+ group had more patients with a poor pathological TNM stage than subgroup A of the N+ group (Table 1). In the N+ group, the positive LN/RLN ratio was 3735/27,408 (13.6%), and the negative LN/RLN ratio was 23 673/27,408 (86.4%). During the subgroup analysis, the positive LN/RLN ratio was 1,355/8136 (16.7%), and the negative LN/RLN ratio was 6781/8,136 (83.3%) in subgroup A of the N+ group. The positive LN/RLN ratio was 2380/19,272 (12.3%), and the negative LN/RLN ratio was 16 892/19,272 (87.7%) in subgroup B of the N+ group, Supplemental Digital Content 2, http://links.lww.com/JS9/A572.

**Table 1:** Demographic characteristics of the N0 and N+ groups.

	N0 group (*n*=641)			N+ group (*n*=983)		
Characteristic	Subgroup A (*n*=347)	Subgroup B (*n*=294)	*P*	Subgroup A (*n*=427)	Subgroup B (*n*=556)	*P*
Sex			0.068			0.291
Male	273 (78.7%)	248 (84.4%)		353 (82.7%)	474 (85.3%)	
Female	74 (21.3%)	46 (15.6%)		74 (17.3%)	82 (14.7%)	
Age, years						
Median (range)	63.0 (37–82)	62.0 (37–85)	0.448	63.0 (35–79)	61.0 (39–82)	0.327
<75	329 (94.8%)	283 (96.3%)		407 (95.3%)	537 (96.6%)	
≥75	18 (5.2%)	11 (3.7%)		20 (4.7%)	19 (3.4%)	
Pathologic differentiation grade			0.026			0.599
Well G1	79 (22.8%)	78 (26.5%)		60 (14.1%)	75 (13.5%)	
Moderate G2	140 (40.3%)	137 (46.6%)		191 (44.7%)	234 (42.1%)	
Poor or undifferentiated G3	128 (36.9%)	79 (26.9%)		176 (41.2%)	247 (44.4%)	
Lymphovascular invasion			0.183			0.206
Yes	32 (9.2%)	18 (6.1%)		111 (26.0%)	125 (22.5%)	
No	315 (90.8%)	276 (93.9%)		316 (74.0%)	431 (77.5%)	
Nerve invasion			0.661			0.184
Yes	51 (14.7%)	47 (16.0%)		101 (23.7%)	111 (20.0%)	
No	296 (85.3%)	247 (84.0%)		326 (76.3%)	445 (80.0%)	
Tumor location			0.03			0.079
Upper	73 (21.0%)	84 (28.6%)		91 (21.3%)	142 (25.5%)	
Middle	208 (59.9%)	147 (50.0%)		219 (51.3%)	293 (52.7%)	
Lower	66 (19.0%)	63 (21.4%)		117 (27.4%)	121 (21.8%)	
Pathological T stage			0.005			0.998
T1b	50 (14.4%)	21 (7.1%)		20 (4.7%)	26 (4.7%)	
T2	87 (25.1%)	66 (22.4%)		82 (19.2%)	106 (19.1%)	
T3	210 (60.5%)	207 (70.4%)		325 (76.1%)	424 (76.3%)	
TNM stage			0.017			0.006
I	75 (21.6%)	42 (14.3%)		0	0	
II	272 (78.4%)	252 (85.7%)		16 (3.7%)	17 (3.1%)	
III	0	0		361 (84.5%)	432 (77.7%)	
IV	0	0		50 (11.7%)	107 (19.2%)	
Thoracic surgery			0.031			0.063
MIE, *n* (%)	203 (58.5%)	147 (50.0%)		202 (47.3%)	230 (41.4%)	
OE, *n* (%)	144 (41.5%)	147 (50.0%)		225 (52.7%)	326 (58.6%)	
Abdominal surgery			0.099			0.220
MIE, *n* (%)	163 (47.0%)	119 (40.5%)		166 (38.9%)	195 (35.1%)	
OE, *n* (%)	184 (53.0%)	175 (59.5%)		261 (61.1%)	361 (64.9%)	
Clinical treatment modality			0.572			0.743
Surgery alone	212 (61.1%)	183 (62.2%)		182 (42.6%)	252 (45.3%)	
Surgery plus postoperative CT or RT/CRT	135 (38.9%)	111 (37.8%)		245 (57.4%)	304 (54.7%)	

CRT, chemoradiotherapy; CT, chemotherapy; MIE, minimally invasive esophagectomy; OE, open esophagectomy; RT, radiotherapy.

### Overall survival

**Figure 2:**
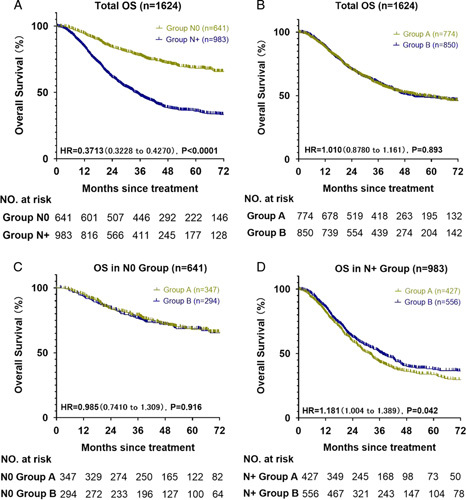
Overall survival curves of participants. (A) Overall survival curve of the N0 and N+ groups. (B) Overall survival curve of subgroup A (RLNs: 15–23) and subgroup B (RLNs: ≥24). (C) Overall survival curve of subgroups A and B of the N0 group. (D) Overall survival curve of subgroups A and B of the N+ group. RLN, resected lymph node.

After a median follow-up of 60.3 months, the median OS of the 1624 patients was 52.3 months (95% CI: 42.4–62.2 months), and that of the N+ group was 33.9 months (95% CI: 30.7–37.1 months). However, the N0 group did not achieve the median OS. The OS rates at 1, 3, and 5 years were 94, 78, and 68%, respectively, in the N0 group. In the N+ group, the OS rates at 1, 3, and 5 years were 84, 47, and 36%, respectively (HR: 0.371; 95% CI: 0.323–0.427; *P*<0.001) (Fig. 2A); there was no significant difference between subgroups A and B (HR: 1.010; 95% CI: 0.741–1.309; *P=*0.893) (Fig. 2B). To explore the effect of LNM on patient survival in the two groups, we performed a subgroup analysis based on the LNM. There was no significant difference between subgroups A and B of the N0 group (HR: 0.985; 95% CI: 0.741–1.309; *P=*0.916) (Fig. 2C). In the N+ group, the median OS of subgroup A was 31.2 months (95% CI: 27.6–34.8 months), and that of subgroup B was 37.1 months (95% CI: 32.3–41.9 months). Furthermore, in the N+ group, the OS rates at 1, 3, and 5 years were 82, 43, and 34%, respectively, in subgroup A; in contrast, they were 86, 51, and 38%, respectively, in subgroup B (HR: 1.181; 95% CI: 1.004–1.389; *P=*0.042) (Fig. 2D).

In this study, there was no significant difference between subgroups A and B regarding the median number of RLNs. These results were confirmed by the N0 group samples. In the N0 group, subgroup A (3-year OS: 79%; 5-year OS: 68%) did not achieve significantly better outcomes than subgroup B (3-year OS: 76%; 5-year OS: 68%; HR: 0.985; *P*=0.916) (Fig. 2C). However, in the N+ group, subgroup B achieved significantly better outcomes (3-year OS: 51%; 5-year OS: 38%; HR: 1.181; *P*=0.042) (Fig. 2D) than subgroup A (3-year OS: 43%; 5-year OS: 34%).

### Risk factors

A single-factor analysis indicated that significant factors that affected OS at 5 years after esophagectomy were sex (*P*<0.001), tumor grade (*P*<0.001), lymphovascular invasion (*P*<0.001), nerve invasion (*P*<0.001), pathological T stage (*P*<0.001), pathological N stage (*P*<0.001), and TNM stage (*P*<0.001) (Table 2). The multifactorial analysis revealed that sex (*P*=0.001), tumor grade (*P*=0.027), lymphovascular invasion (*P*=0.004), nerve invasion (*P*=0.033), TNM stage (*P*<0.001), and T stage (*P*=0.002) were important factors that affected OS at 5 years after esophagectomy (Table 2). However, the number of RLNs did not have a significant effect on the total patient cohort (*P*=0.863).

**Table 2:** Univariate and multivariate cox regression analyses of factors affecting patient survival.

	Univariate			Multivariate		
Variables	HR	95% CI	*P*	HR	95% CI	*P*
Sex						
Male	Ref.				Ref.	
Female	0.634	(0.514–0.781)	<0.001	0.704	(0.57–0.868)	0.001
Age, years						
<75	Ref.					
≥75	1.351	(0.971–1.88)	0.074			
Pathologic differentiation grade			<0.001			0.027
Well G1	Ref.				Ref.	
Moderate G2	1.425	(1.149–1.768)	0.001	1.204	(0.963–1.505)	0.103
Poor or undifferentiated G3	1.666	(1.343–2.068)	<0.001	1.351	(1.078–1.692)	0.009
Lymphovascular invasion						
Yes		Ref.			Ref.	
No	0.548	(0.464–0.647)	<0.001	0.776	(0.652–0.923)	0.004
Nerve invasion						
Yes		Ref.			Ref.	
No	0.671	(0.568–0.794)	<0.001	0.829	(0.698–0.985)	0.033
Tumor location			0.449			
Upper	Ref.					
Middle	1.119	(0.94–1.333)	0.207			
Lower	1.074	(0.872–1.321)	0.503			
Pathological T stage			<0.001			0.002
T1b		Ref.			Ref.	
T2	1.544	(1.053–2.263)	0.026	1.061	(0.619–1.819)	0.83
T3	2.455	(1.73–3.485)	<0.001	1.496	(0.87–2.573)	0.146
Pathological N stage						
N0		Ref.				
N+	2.714	(2.306–3.195)	<0.001	1.051	(0.455–2.427)	0.908
TNM stage			<0.001			<0.001
I		Ref.			Ref.	
II	1.576	(1.037–2.397)	0.033	1.056	(0.6–1.858)	0.851
III	3.764	(2.517–5.631)	<0.001	2.269	(0.704–7.309)	0.17
IV	7.152	(4.652–10.994)	<0.001	3.778	(1.161–12.287)	0.027
Thoracic surgery						
MIE		Ref.				
OE	1.07	(0.997–1.148)	0.06			
Abdominal surgery						
MIE		Ref.				
OE	1.07	(0.994–1.151)	0.07			
Clinical treatment modality						
Surgery alone		Ref.				
Surgery plus postoperative CT or RT/CRT	1.038	(0.968–1.113)	0.293			
Surgical techniques			0.683			
Two-field		Ref.				
Three-field	0.897	(0.645–1.249)	0.521			
Unilateral neck and two-field	1.111	(0.771–1.601)	0.573			

CRT, chemoradiotherapy; CT, chemotherapy; HR, hazard ratio; MIE, minimally invasive esophagectomy; OE, open esophagectomy; RT, radiotherapy.

## Discussion

**Figure 3:**
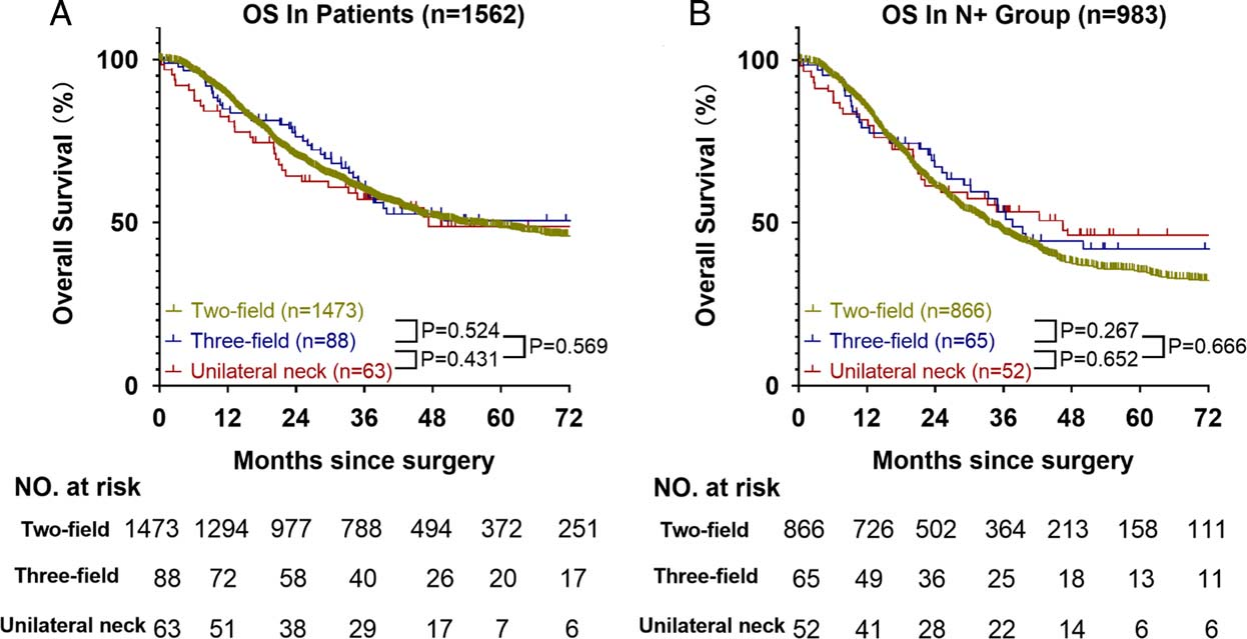
Overall survival curves of participants subjected to different surgical techniques. (A) Overall survival curve of the total patients. (B) Overall survival curve of patients in the N+ group.

This study clarified the value of RLNs during lymphadenectomy in terms of long-term survival benefits. Compared with the N0 group, the N+ group exhibited significantly improved OS as the number of RLNs increased. In the N+ group, the OS of subgroup B was better than that of subgroup A, and there were no statistically significant differences in the demographic characteristics between groups, except for the TNM stage and surgical techniques for lymphadenectomy. However, in the N+ group, the number of cases worse than TNM stage IV was higher in subgroup B (19.2%) than in subgroup A (11.7%) (*P*=0.006). Although the surgical techniques for esophageal carcinoma were not the same in terms of LN harvesting, surgical techniques for lymphadenectomy had no statistical significance in the single-factor analysis (Table 2). Moreover, the three groups of patients were very close in the Kaplan–Meier curves, with a nonsignificant *P*-value in total patients and the N+ group (Fig. 3). Sex, tumor grade, T stage, and N stage significantly influenced the OS of patients in this study (Table 3).

**Table 3:** Univariate and multivariate cox regression analyses of factors affecting survival of patients in the N+ group.

	Univariate			Multivariate		
Variables	HR	95% CI	*P*	HR	95% CI	*P*
Sex						
Male	Ref.				Ref.	
Female	0.661	(0.517–0.844)	0.001	0.658	(0.514–0.842)	0.001
Age, years						
<75	Ref.					
≥75	1.679	(1.156–2.439)	0.006	1.882	(1.293–2.74)	0.001
Pathologic differentiation grade			0.068			
Well G1	Ref.					
Moderate G2	1.219	(0.939–1.584)	0.137			
Poor or undifferentiated G3	1.351	(1.042–1.753)	0.023			
Lymphovascular invasion						
Yes		Ref.			Ref.	
No	0.626	(0.524–0.749)	<0.001	0.644	(0.537–0.771)	<0.001
Nerve invasion						
Yes		Ref.			Ref.	
No	0.754	(0.624–0.912)	0.004	0.878	(0.723–1.066)	0.189
Tumor location			0.201			
Upper	Ref.					
Middle	1.195	(0.975–1.465)	0.086			
Lower	1.081	(0.852–1.371)	0.521			
Pathological T stage			<0.001			<0.001
T1b		Ref.			Ref.	
T2	1.408	(0.86–2.307)	0.174	1.465	(0.893–2.401)	0.13
T3	2.154	(1.362–3.408)	0.001	2.077	(1.309–3.296)	0.002
TNM stage			<0.001		–	
II		Ref.			–	
III	0.174	(0.088–0.342)	<0.001		–	
IV	0.528	(0.433–0.643)	<0.001		–	
Thoracic surgery						
MIE		Ref.				
OE	1.058	(0.975–1.148)	0.176			
Abdominal surgery						
MIE		Ref.				
OE	1.062	(0.975–1.156)	0.167			
The number of LN resection						
23–24		Ref.				
>24	0.847	(0.721–0.995)	0.043	0.859	(0.731–1.009)	0.065
Clinical treatment modality						
Surgery alone		Ref.				
Surgery plus postoperative CT or RT/CRT	0.964	(0.889–1.046)	0.379			
Surgical techniques			0.502			
Two-field		Ref.				
Three-field	0.817	(0.572–1.167)	0.266			
Unilateral neck and two-field	0.92	(0.629–1.345)	0.665			

CRT, chemoradiotherapy; CT, chemotherapy; HR, hazard ratio; MIE, minimally invasive esophagectomy; OE, open esophagectomy; RT, radiotherapy.

These findings suggest that the treatment strategy should depend on the presence of LNM. For patients without LNM, there was no significant difference in OS between patients with 15–23 LNs and those with 24 or more LNs dissected during surgery. Therefore, the current Chinese expert consensus and guidelines, which indicate that the required number of resected LNs should exceed 15, are reasonable^4^. However, for patients with LNM, the surgical requirements described in the Chinese Society of Clinical Oncology diagnosis and treatment guidelines for malignant LNs in 2021 require further consideration. In our study, patients with LNM who underwent the removal of at least 24 LNs exhibited considerably better OS than patients who underwent the removal of 15–23 LNs. This suggests that the surgical strategy for patients with suspected LNM should include the removal of at least 24 LNs during esophagectomy. The OS of patients with N+ can be considerably improved by increasing the number of LNs removed during surgery to 24 or more. Therefore, this is a favorable surgical treatment strategy. Moreover, the clinical stage of patients should be carefully distinguished before esophagectomy, especially when there is suspicion of LNM indicated by computed tomography, ultrasonography, or positron emission tomography^16,17^, and the surgical treatment strategy should be adjusted appropriately^18,19^.

The Memorial Sloan–Kettering Cancer Center proposed that 5-year OS is dependent on the T classification: for pT1, the resection of 10 LNs is adequate; for pT2, at least 20 LNs should be removed; and for pT3/T4, 30 or more LNs should be removed^20^. Similarly, different pN stages require the removal of different LNs. A study performed at the Cleveland Clinic suggested that the removal of at least 25 LNs was optimal for patients receiving neoadjuvant therapy for esophageal adenocarcinoma^20^, and our study showed no obvious difference in the OS of subgroups A and B. This indicates that the OS of patients did not change much after the removal of a sufficient number of LNs. However, LNM is an independent risk factor for OS; therefore, we individually evaluated the LNM of each patient. In this study, the number of LNs removed during lymphadenectomy was associated with significantly improved OS for TESCC patients with LNM. We hope to provide more evidence regarding the characteristics and surgical treatment of TESCC for medical professionals in China. Concerning the clinical efficacy of LN removal at each station for TESCC patients, further studies including more samples and clinical data from more centers are required to verify these findings and improve the OS of patients with esophageal cancer.

In recent years, various studies, such as CROSS, NEOCRTEC5010, and CheckMate-577, of the treatment of esophageal cancer have attracted considerable attention, and a comprehensive treatment modality based on esophagectomy was formulated. The OS of patients was significantly different in the NEOCRTEC5010 and CROSS trials; however, LN dissection might have affected these results, especially those of patients with suspected LNM^20–25^. Metastases of vestigial LNs could negatively impact OS and reduce the effects of subsequent treatment. We deduced from our results that the removal of at least 24 LNs is crucial to the surgical treatment of patients with N+.

Unfortunately, the relevant lymphadenectomy guidelines and consensus for patients with suspected LNM do not adequately apply to all patients. When patients have suspiciously large LNs, it is sufficient to remove 15 or more LNs; however, for patients with N0, some stations do not require consideration in accordance with lymphadenectomy standards. Conversely, a careful and systematic evaluation of each LN station is vital for an accurate and appropriate lymphadenectomy. Therefore, these challenges must be urgently addressed.

There are some limitations to our study. This study did not consider real-world confounding factors that could have influenced the results. Furthermore, 12 groups at our center performed esophagectomy from January 2010 to December 2017. McKeown esophagectomy and Ivor–Lewis esophagectomy were the main surgical types, two-field or three-field lymphadenectomy was performed, and careful systematic lymphadenectomy was not performed at each station. Therefore, certain subjective selection bias exists in the results. The clinical value and efficacy index of different lymph stations were different. Our research of the efficacy index will be presented in the near future. This study included only single-center data that were retrospectively collected; therefore, their generalizability requires consideration. Moreover, the results of postoperative complications were lacking in this study, and only the OS outcomes were evaluated. Multicenter cooperation among hospitals in China is required to obtain larger cohorts to collect compelling evidence to enhance the guidelines for lymphadenectomy for esophageal cancer. By combining the results of multicenter data analyses, specific and detailed treatment options can be developed.

## Conclusions

Increasing the number of LNs harvested during surgery to 24 or more could improve the OS of patients with N+. Patients without LNM achieve better OS than those with LNM. The guidelines recommend dissecting at least 15 LNs for patients without LNM, and no further OS benefit was observed when the number of harvested LNs was increased for these patients. Patients with different stages of disease should be provided with different LN dissection strategies.

## Ethical approval

All procedures performed in this study were in accordance with the Declaration of Helsinki (as revised in 2013). The study was approved by the Ethics Committee (EC) for Medical Research and New Medical Technology of Sichuan Cancer Hospital (SCCHEC-02-2022-050). Consent was waived by the Ethics Committee (EC) due to the retrospective nature of the study.

## Funding

The study was supported by grants from the National Key Research and Development Program (2022YFC2403400), International Cooperation Projects of Science and Technology Department of Sichuan Province (Grant No. 2020YFH0169), the Sichuan Key Research and Development Project from Science and Technology Department of Sichuan Province (Grant No. 2023YFS0044, 2023YFQ0055, 2023YFQ0056, No. 2021YJ0118), the Wu Jieping Clinical Research Projects (Grant No. 320.6750.2020-15-3), and Sichuan Province Clinical Key Specialty Construction Project.

## Author contribution

All authors contributed in the study concept and design, acquisition, analysis, or interpretation of data. K.L.: drafting of the article and statistical analysis; L.P.: administrative, technical, or material support and obtained funding; Y.H.: study supervision. All authors contributed in revising the article critically for important intellectual content and final approval of the version to be published. K.L., X.L., and W.H. had full access to all the data in the study and take responsibility for the integrity of the data and the accuracy of the data.

## Conflicts of interest disclosure

None.

## Research registration unique identifying number (UIN)

1. Name of the registry: ClinicalTrials.gov.
2. Unique Identifying number or registration ID: NCT05570487.
3. Hyperlink to your specific registration (must be publicly accessible and will be checked): https://clinicaltrials.gov/ct2/show/NCT05570487?term=NCT05570487&draw=2&rank=1.

## Guarantor

Kexun Li and Lin Peng are guarantors.

## Data availability statement

We are willing to share data, analytic methods, and study materials related to this article with other researchers, provided that all of the above will not be used for commercial or profit purposes. Other researchers can contact the corresponding author of this article by e-mail and indicate the required research materials and purpose. We will gladly provide relevant materials for this study after approval and discussion.

## Provenance and peer review

Not commissioned, externally peer-reviewed.

## Supplementary Material

## References

[1] Kocarnik JM Compton K Dean FE . Cancer incidence, mortality, years of life lost, years lived with disability, and disability-adjusted life years for 29 cancer groups from 2010 to 2019: a systematic analysis for the Global Burden of Disease Study 2019. JAMA Oncol 2022;8:420–444. 34967848 10.1001/jamaoncol.2021.6987 PMC8719276

[2] Sung H Ferlay J Siegel RL . Global cancer statistics 2020: GLOBOCAN estimates of incidence and mortality worldwide for 36 cancers in 185 countries. CA Cancer J Clin 2021;71:209–249. 33538338 10.3322/caac.21660

[3] Liang H Fan JH Qiao YL . Epidemiology, etiology, and prevention of esophageal squamous cell carcinoma in China. Cancer Biol Med 2017;14:33–41. 28443201 10.20892/j.issn.2095-3941.2016.0093 PMC5365188

[4] Wang FH Zhang XT Li YF . The Chinese Society of Clinical Oncology (CSCO): clinical guidelines for the diagnosis and treatment of gastric cancer, 2021. Cancer Commun (Lond) 2021;41:747–795. 34197702 10.1002/cac2.12193 PMC8360643

[5] Shapiro J Van Lanschot JJ Hulshof MC . Neoadjuvant chemoradiotherapy plus surgery versus surgery alone for oesophageal or junctional cancer (CROSS): long-term results of a randomised controlled trial. Lancet Oncol 2015;16:1090–1098. 26254683 10.1016/S1470-2045(15)00040-6

[6] Kelly RJ Ajani JA Kuzdzal J . Adjuvant nivolumab in resected esophageal or gastroesophageal junction cancer. N Engl J Med 2021;384:1191–1203. 33789008 10.1056/NEJMoa2032125

[7] Hagens ER van Berge Henegouwen MI Van Sandick JW . Distribution of lymph node metastases in esophageal carcinoma [TIGER study]: study protocol of a multinational observational study. BMC Cancer 2019;19:1–8. 31272485 10.1186/s12885-019-5761-7 PMC6610993

[8] Li B Zhang Y Miao L . Esophagectomy with three-field versus two-field lymphadenectomy for middle and lower thoracic esophageal cancer: long-term outcomes of a randomized clinical trial. J Thorac Oncol 2021;16:310–317. 33307192 10.1016/j.jtho.2020.10.157

[9] Altorki N Kent M Ferrara C . Three-field lymph node dissection for squamous cell and adenocarcinoma of the esophagus. Ann Surg 2002;236:177–183. 12170022 10.1097/00000658-200208000-00005 PMC1422563

[10] Okholm C Svendsen LB Achiam MP . Status and prognosis of lymph node metastasis in patients with cardia cancer – a systematic review. Surg Oncol 2014;23:140–146. 24953457 10.1016/j.suronc.2014.06.001

[11] Stein HJ Feith M Bruecher BL . Early esophageal cancer: pattern of lymphatic spread and prognostic factors for long-term survival after surgical resection. Ann Surg 2005;242:566–573. 16192817 10.1097/01.sla.0000184211.75970.85 PMC1402356

[12] Twine CP Lewis WG Morgan MA . The assessment of prognosis of surgically resected oesophageal cancer is dependent on the number of lymph nodes examined pathologically. Histopathology 2009;55:46–52. 19614766 10.1111/j.1365-2559.2009.03332.x

[13] Yu L Zhang XT Guan SH . The number of negative lymph nodes is positively associated with survival in esophageal squamous cell carcinoma patients in China. Open Med (Wars) 2020;15:152–159. 32190739 10.1515/med-2020-0023 PMC7065439

[14] Li B Hu H Zhang Y . Esophageal squamous cell carcinoma patients with positive lymph nodes benefit from extended radical lymphadenectomy. J Thorac Cardiovasc Surg 2019;157:1275–1283. 33198003 10.1016/j.jtcvs.2018.11.094

[15] Agha R Abdall-Razak A Crossley E . The STROCSS 2019 guideline: strengthening the reporting of cohort studies in surgery. Int J Surg 2019;72:156–165. 31704426 10.1016/j.ijsu.2019.11.002

[16] Choi J Kim SG Kim JS . Comparison of endoscopic ultrasonography (EUS), positron emission tomography (PET), and computed tomography (CT) in the preoperative locoregional staging of resectable esophageal cancer. Surg Endosc 2010;24:1380–1386. 20033712 10.1007/s00464-009-0783-x

[17] Onbaş O Eroglu A Kantarci M . Preoperative staging of esophageal carcinoma with multidetector CT and virtual endoscopy. Eur J Radiol 2006;57:90–95. 16122893 10.1016/j.ejrad.2005.07.012

[18] Yuan F Qingfeng Z Jia W . Influence of metastatic status and number of removed lymph nodes on survival of patients with squamous esophageal carcinoma. Medicine 2015;94:e1973. 26632887 10.1097/MD.0000000000001973 PMC4674190

[19] Wang YX Wang LL Yang Q . Impact of number of dissected lymph nodes on survival in patients with thoracic esophageal squamous cell carcinoma after radical resection. [Chinese]. Zhonghua Zhong Liu Za Zhi 2016;38:150–155. 26899337 10.3760/cma.j.issn.0253-3766.2016.02.014

[20] Rizk NP Ishwaran H Rice TW . Optimum lymphadenectomy for esophageal cancer. Ann Surg 2010;251:46–50. 20032718 10.1097/SLA.0b013e3181b2f6ee

[21] Terada M Hara H Daiko H . Phase III study of tri-modality combination therapy with induction docetaxel plus cisplatin and 5-fluorouracil versus definitive chemoradiotherapy for locally advanced unresectable squamous-cell carcinoma of the thoracic esophagus (JCOG1510: TRIANgLE). Jpn J Clin Oncol 2019;49:1055–1060. 31411696 10.1093/jjco/hyz112

[22] Visser E Edholm D Smithers BM . Neoadjuvant chemotherapy or chemoradiotherapy for adenocarcinoma of the esophagus. J Surg Oncol 2018;117:1687–1696. 29806960 10.1002/jso.25089

[23] Li C Zhao S Zheng Y . Preoperative pembrolizumab combined with chemoradiotherapy for oesophageal squamous cell carcinoma (PALACE-1). Eur J Cancer 2021;144:232–241. 33373868 10.1016/j.ejca.2020.11.039

[24] Leng X He W Yang H . Prognostic impact of postoperative lymph node metastases after neoadjuvant chemoradiotherapy for locally advanced squamous cell carcinoma of esophagus: from the results of NEOCRTEC5010, a randomized multicenter study. Ann Surg 2021;274:e1022–e1029. 31855875 10.1097/SLA.0000000000003727

[25] Shen J Kong M Yang H . Pathological complete response after neoadjuvant treatment determines survival in esophageal squamous cell carcinoma patients (NEOCRTEC5010). Ann Transl Med 2021;9:1516. 34790722 10.21037/atm-21-3331 PMC8576689

