# Supplementary material for: Resected lymph nodes and survival of patients with esophageal squamous cell carcinoma: an observational study
Source: Int J Surg. 2023 May 24;109(7):2001–9. doi: 10.1097/JS9.0000000000000436 (PMC10389544; doi:10.1097/JS9.0000000000000436)
Supplement: Supplementary file 2 [file js9-109-2001-s002.docx]

**Supplementary Table S1**. The positive and negative nodes in each group.

| Pathology Results | N+ group (n = 983) | | |
| --- | --- | --- | --- |
|  | Subgroup A  (n = 427) | Subgroup B  (n = 556) | Total  (n = 983) |
| LNs |  |  |  |
| Positive (%) | 1,355  (16.7%) | 2,380  (12.3%) | 3,735  (13.6%) |
| Negative (%) | 6,781  (83.3%) | 16,892  (87.7%) | 23,673  (86.4%) |

LNs, lymph nodes.
